# Chromomycin A2 Induces Autophagy in Melanoma Cells

**DOI:** 10.3390/md12125839

**Published:** 2014-12-04

**Authors:** Larissa Alves Guimarães, Paula Christine Jimenez, Thiciana da Silva Sousa, Hozana Patrícia S. Freitas, Danilo Damasceno Rocha, Diego Veras Wilke, Jesús Martín, Fernando Reyes, Otília Deusdênia Loiola Pessoa, Letícia Veras Costa-Lotufo

**Affiliations:** 1 Marine Sciences Institute, Federal University of Ceara, Fortaleza, Ceara 60165-081, Brazil; E-Mail: larissaalvesgui@gmail.com; 2 Department of Physiology and Pharmacology, Federal University of Ceara, Fortaleza, Ceará 60430-270, Brazil; E-Mails: danilodrocha@yahoo.com.br (D.D.R.); diegowilke@gmail.com (D.V.W.); 3 Marine Sciences Department, Federal University of São Paulo, Santos, São Paulo 11030-400, Brazil; E-Mail: paulacjimenez@gmail.com; 4 Department of Organic and Inorganic Chemistry, Federal University of Ceara, Fortaleza, CE 60021-970, Brazil; E-Mails: thicy85@yahoo.com.br (T.S.S.); hpatriciasf@yahoo.com.br (H.P.S.F.); opessoa@ufc.br (O.D.L.P.); 5 Fundación MEDINA, Centro de Excelencia en Investigación de Medicamentos Innovadores en Andalucía, Granada 18016, Spain; E-Mails: jesus.martin@medinaandalucia.es (J.M.); fernando.reyes@medinaandalucia.es (F.R.)

**Keywords:** *Streptomyces*, autophagy, cytotoxicity, chromomycin, marine microorganisms, Brazil

## Abstract

The present study highlights the biological effects of chromomycin A2 toward metastatic melanoma cells in culture. Besides chromomycin A2, chromomycin A3 and demethylchromomycin A2 were also identified from the extract derived from *Streptomyces* sp., recovered from Paracuru Beach, located in the northeast region of Brazil. The cytotoxic activity of chromomycin A2 was evaluated across a panel of human tumor cell lines, which found IC_50_ values in the nM-range for exposures of 48 and 72 h. MALME-3M, a metastatic melanoma cell line, showed the highest sensitivity to chromomycin A2 after 48h incubation, and was chosen as a model to investigate this potent cytotoxic effect. Treatment with chromomycin A2 at 30 nM reduced cell proliferation, but had no significant effect upon cell viability. Additionally, chromomycin A2 induced accumulation of cells in G_0_/G_1_ phase of the cell cycle, with consequent reduction of S and G_2_/M and unbalanced expression of cyclins. Chromomycin A2 treated cells depicted several cellular fragments resembling autophagosomes and increased expression of proteins LC3-A and LC3-B. Moreover, exposure to chromomycin A2 also induced the appearance of acidic vacuolar organelles in treated cells. These features combined are suggestive of the induction of autophagy promoted by chromomycin A2, a feature not previously described for chromomycins.

## 1. Introduction

Chromomycins are glycosylated tricyclic polyketides members of the aureolic acid group of antitumor antibiotics. These golden-yellow compounds are produced by actinomycetes, mainly those from the *Streptomyces* genus. Although chromomycins were initially isolated due to their antibacterial activity, the main pharmacological interest in such molecules regards their anticancer activity [1].

Mithramycin was the first compound described from this class, in the early 1950s, and its production has been associated with *Streptomyces argillaceus*, *S. plicatus*, *S. tanashiensis* and *S. atroolivaceus* [1,2]. Clinically, it has been used to treat certain types of cancer, such as testicular carcinoma, and Paget’s disease [3,4,5]. In addition, lately, this compound has been finding new therapeutic uses in the treatment of cancer and other diseases [6].

Chromomycin A3, the best-known compound among the chromomycins, was isolated in 1958 from the fermentation broth of *S. griseus* [7,8]. It is a very potent inhibitor of tumor cell growth and also shares some bioactivities with mithramycin, such as inhibition of neuronal apoptosis [9] and induction of erythroid differentiation in K562 cells [10]. However, the clinical use of these compounds has been limited due to toxicity towards liver, kidneys and gastrointestinal organs [11].

Several studies have shown that the antitumor effect of chromomycins and other aureolic acids is partly owned to non-intercalating interaction with G-C regions in the minor grove of the DNA double helix, by forming dimeric complexes with the Mg^2+^. This interaction affects the processes of replication and, mainly, transcription of DNA, and is determinant for the cytotoxic activity [12,13].

The chromomycin studied herein was isolated from a strain of *Streptomyces* sp. recovered from sediment collected at Paracuru Beach, located on the west coast of Ceará State, on the northeastern region of Brazil. This study is part of a larger project, which is comprised of the biotechnological prospection of marine bacteria associated to sediments from the tropical coast of Brazil by means of dereplication of cytotoxic extracts and isolation of bioactive compounds. A further mode of action evaluation also constitutes a goal of the project. Therefore, the present article, one of the first originated within this venture, reports on evidences that show the induction of autophagy in melanoma cells by chromomycin A2, a feature not yet described for this class of compounds.

## 2. Results and Discussion

**Figure 1:**
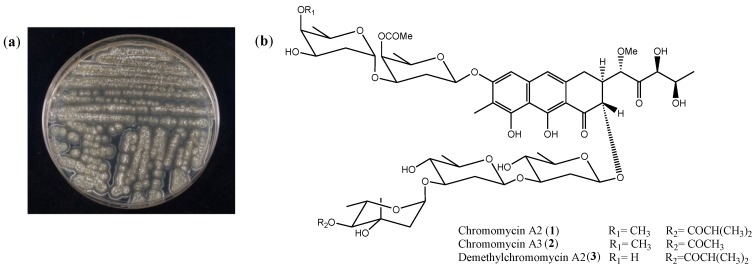
Bacterial strain and chemical structure. (**a**) Photograph of *Streptomyces* sp. plated on solid A1 media; (**b**) Structures of chromomycin A2 (**1**) [21,22], chromomycin A3 (**2**) [7,8] and demethylchromomycin A2 (**3**) [23,24].

Actinomycetes are Gram-positive bacteria widely distributed in soil from terrestrial and aquatic environments. These microorganisms play an essential role in decomposition of complex mixtures and recycling of nutrients. Moreover, they are renowned factories of bioactive secondary metabolites, including those of clinical significance, and account for the biosynthesis of nearly 40% known microbial natural products [14,15]. Within these producers of bioactive compounds, members of their largest genus, *Streptomyces*, deserve special consideration since they are responsible for making around 75% of all actinomycete-derived bioactive compounds [16], and 60% of clinically valuable antibiotics [17]. The *Streptomyces* sp. strain studied herein (Figure 1a) was selected based on a cell based screening using MTT assay, in which its EtOAc extract presented cytotoxicity towards the adenocarcinoma HCT-116 cell line in the sub-microgram range, and was identified based on the 16S sequencing and Basic Local Alignment Search Tool (BLAST) analysis.

### 2.1. Isolation and Cytotoxicity of Chromomycin A2

The active EtOAc extract of *Streptomyces* sp. was fractionated using reversed phase flash chromatography, resulting in 15 fractions (F1–F15). LC-HRESIMS analysis of fraction F8 revealed the presence of peaks with pseudomolecular ions at *m/z* ([M + H]^+^) of 1211.5476, (calcd. for C_59_H_87_O_26_, 1211.5480), 1183.5164 (calcd. for C_57_H_83_O_26_, 1183.5167) and 1197.5310 (calcd. for C_58_H_85_O_26_, 1197.5324), corresponding to the known compounds chromomycin A2 (**1**), chromomycin A3 (**2**) and demethylchromomycin A2 (**3**) (Figure 1b), respectively. Fraction F8 was chromatographed on a reversed phase column using a gradient H_2_O/CH_3_CN and the subfractions were finally purified by repeated semipreparative and preparative HPLC to yield 1.0 mg of the major compound **1** as a yellow solid and negligible amounts of compounds **2** and **3**. Compound **1** was identified as chromomycin A2, by means of HRMS and ^1^H NMR spectroscopic data (Supplementary Figures S1 and S2) and comparison with previously published data [18,19,20].

Chromomycin A2 (**1**), also known as aburamycin A, was firstly isolated in 1957 [21,22] from *S. aburaviensis* and, since then, it has been reported to be produced by other *Streptomyces* species [25,26,27]. Chromomycin A2, differently from chromomycin A3, has not been extensively studied concerning its biological activity. It has been shown, however, that chromomycin A2 may be up to 10 times more cytotoxic than chromomycin A3 [11,28] and both compounds are active within the low nM-range. Demethylchromomycin A2 was also isolated from *S. aburaviensis* in 1988, and presented inhibitory activity against gram-positive bacteria and towards the P-388 leukemia model in mice, but it was less active then chromomycin A3 in the experimental cancer model [23,24].

On the 7-cell line panel of the present study, chromomycin A2 also induced cytotoxicity in the nM-range after 48 or 72 h incubation (Table 1). Interestingly, exposure of cells to chromomycin A2 during a shorter period, for example, 24 h, was insufficient to induce a notable effect on cell proliferation, even at concentrations 20 to 60 times higher than the IC_50_ calculated for cells exposed during longer periods (72 h). Chromomycin A2 was slightly selective towards cancer cells when compared to non-tumor cell line MRC-5. The obtained selective index after 72 h incubation (IC_50_normal cells/IC_50_ tumor cells) ranged from 2.2 in metastatic prostate PC-3M cells to 17 in adenocarcinoma HCT-116 cells. Lu *et al.* [28] evaluated the cytotoxicity of chromomycin A2 against the human umbilical vein endothelial cells (HUVEC) and obtained IC_50_ values of 8 nM, much lower than the one obtained in the present study for the fetal lung fibroblast MRC-5, although for the adenocarcinoma cells HCT-116, the IC_50_ value was 5 nM, comparable to the one obtained herein (6.3 nM, Table 1). Thus, it can be suggested that cytotoxicity is variable towards non-tumor cells of different origins, such as observed with tumor cells. Doxorubicin was used as positive control and showed IC_50_ values ranging from 6 nM, for HL-60 leukemia cells, to 3280 nM, for fetal lung fibroblast MRC-5, after 72 h incubation (Supplementary Table S1).

**Table 1:** Cytotoxicity of chromomycin-A2 (**1**) on select cell lines. Activity of **1** was evaluated using the MTT assay after 24, 48 and 72 h incubation. The IC_50_ [nM] values and 95% CI were obtained via nonlinear regression. n.d.—not determined.

Cell Line	Histological Origin (Duplication Time)	IC_50_ [nM] (CI 95%)		
		24 h	48 h	72 h
HCT-116	Colon adenocarcinoma (17.4 h)	>400	21.6 (12.1–38.5)	6.3 (4.6–8.7)
HL-60	Promyelocitic leukemia (28.6 h)	>200	22.3 (19.7–25.3)	10.1 (8.13–12.46)
MALME-3M	Metastatic melanoma (46.2 h)	>400	16.7 (7.2–38.5)	18.7 (14.6–24.3)
OVCAR-8	Ovarian adenocarcinoma (26.1 h)	>200	33.8 (28.2–40.5)	8.8 (7.7–9.9)
PC-3M	Metastatic prostate carcinoma (20 h)	>400	209.8 (122.5–359.5)	49.0 (27.5–87.3)
SF-295	Glioblastoma (29.5 h)	>200	n.d.	20.8 (18.8–23.0)
MRC-5	Lung fibroblast (27 h)	> 400	131.7 (86.6–200.3)	109.0 (82.0–144.8)

The malignant melanoma cell MALME-3M was among the most sensitive cell lines to chromomycin A2, and thus was chosen to further evaluate the mechanisms underlying chromomycin A2 cytotoxic effects. Malignant melanoma, classified by genetic defects within pigment-producing melanocytes, is attributed to the largest number of skin related cancer deaths [29]. While protective measures can be taken, the aggressive and rapid metastatic properties of melanoma-based tumors continues to challenge clinical practices [29]. This, combined with unique epidemiological concerns [30] and lack of viable treatment options [31], places melanoma among the most fatal cancers. Although the cytotoxicity of chromomycin A2 was comparable to doxorubicin for most cancer cells, MALME-3M was around 13 times more sensitive to chromomycin A2 than to the positive control, whereas, in PC-3M, doxorubicin was almost 50 times more active than chromomycin A2. Additionally, herein, chromomycin A3 (isolation and identification procedures described in supplementary material) was also assayed against MALME-3M under the same conditions (48 h incubation) and presented an IC_50_ value of 762 nM, which represents a 45 times decrease in cytotoxicity when compared to chromomycin A2. It was not possible to obtain enough material of demethylchromomycin A2 to run the biological assays.

The potent cytotoxic activity of chromomycins, mithramycin and other structurally similar members of the aureolic acids arises from H-bonds created between the aglycon moiety and guanine bases from the minor grove of the DNA double-helix [2]. The sugar subunits, on the other hand, partake in this interaction by stabilizing the complex compound-DNA and influencing the sequence specificity with which the molecule will bind to DNA [32,33]. Mithramycin and chIromomycins share the same aglycon, however, the slight differences among their glycosidic structures affect the plasticity of their interaction with DNA. The added methyl and acetyl groups on chromomycin A3 (**2**) makes binding more stable and specific when compared to that of mithramycin [34].

To the best of our knowledge, chromomycin A2 has not been directly experimented in a DNA-binding biological model, such as chromomycin A3 and mithramycin [35,36]. It is, however, expected that DNA-binding patterns of both chromomycins be comparable. Nevertheless, differently from both chromomycin A3 and mithramycin, chromomycin A2 carries an extra acetyl group on one of the sugar units, rendering that interaction with DNA, by analogy at least, more stable than that with **2**.

Thus, we decided to further evaluate the mechanisms underlying chromomycin A2 effects on MALME-3M using two concentrations, 10 and 30 nM, that comprised the obtained IC_50_ value (16.7 nM), and an incubation time of 48 h compatible to the doubling time for this cell line (46.2 h). As positive controls, we used doxorubicin (100 nM), a natural anthracycline currently used in cancer chemotherapy [37], and rapamycin (30 nM), a natural macrolide currently used as an immunosuppressant drug and a typical autophagy reference compound for *in vitro* studies [38]. Chromomycin A3 was also included in the analysis to compare its activity profile with that found for chromomycin A2.

### 2.2. Chromomycin A2 Alters Cell Cycle of Metastatic Melanoma Cells MALME-3M

**Figure 2:**
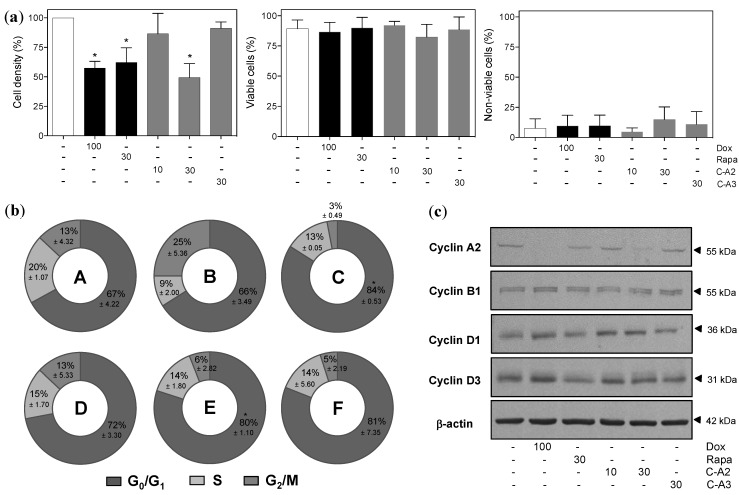
Effect of chromomycin-A2 (**C**-**A2**) on cell viability and cell cycle progression of MALME-3M cells treated with controls, chromomycin A2 at 10 and 30 nM or chromomycin A3 at 30 nM for 48 h. (**a**) Flow cytometry analysis of cell density and cell viability with propidium iodide staining; (**b**) Doughnut graphs depicting distribution of cell cycle phases (A, DMSO; B, doxorubicin; C, rapamycin; D, chromomycin A2 10 nM; E, chromomycin A2 30 nM; F, chromomycin A3, **C**-**A3**, 30 nM); (**c**) Expression of key cell cycle regulating proteins by western blotting. DMSO (0.4% v/v) was used as negative control (−) and doxorubicin (100 nM) and rapamycin (30 nM) were used as positive controls. * *p* < 0.05. Data on graphs correspond to the mean ± standard error of the mean from at least three independent experiments.

Initial flow cytometry analysis showed that chromomycin A2 had no influence on cell viability, but significantly reduced cell density at 30 nM, much like the positive controls (Figure 2a and Supplementary Figure S5). Subsequent analysis of the cell cycle revealed exposure of MALME-3M to chromomycin A2 at 30 nM significantly increased accumulation of cells in the G_0_/G_1_ phase (Figure 2b and Supplementary Figure S6). Western blot analysis strengthened this observation by revealing increased expression of cyclins D1 and D3, which are usually highest during G_1_, and reduced expression of cyclins A2 and B1, which are relevant throughout S and G_2_ phases, after treatment with chromomycin A2 at 30 nM (Figure 2c). Chromomycin A3, at the tested concentration 30nM, presented no significant effect on the parameters evaluated.

### 2.3. Alterations in Morphology and Expression of Autophagic Proteins by Chromomycin A2

**Figure 3:**
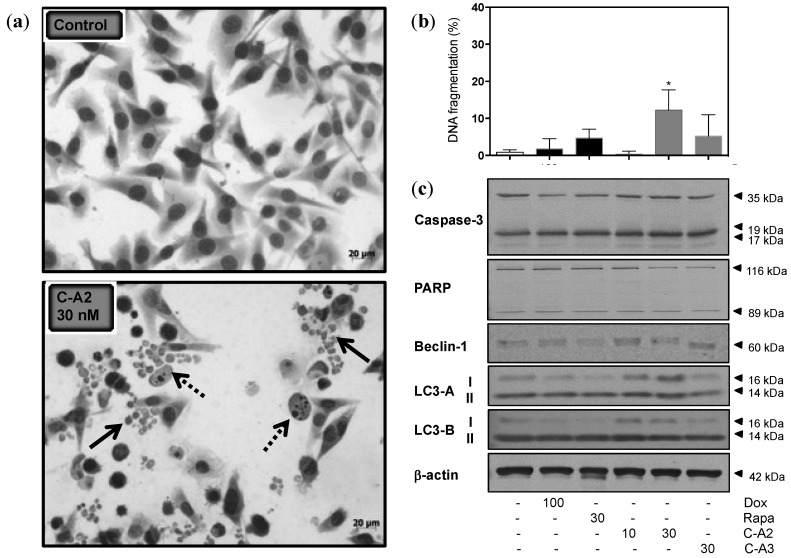
Chromomycin-A2 (**C-A2**) induces alterations in morphology and expression of regulatory proteins of autophagy in MALME-3M cells. (**a**) Photomicrographs of May-Grumwald-Giemsa-stained MALME-3M cells exposed to chromomycin A2 at 30 nM for 48 h in comparison to negative control cells. Arrows indicate the autophagosomes resembling fragments (solid line arrow) and internucleosomal fragmentation (dotted line arrow). (**b**) Flow cytometry analysis of nuclear DNA fragmentation with propidium iodide staining; (**c**) Expression of key apoptosis and autophagy regulating proteins by western blotting. DMSO (0.4% v/v) was used as negative control (−) and doxorubicin (100 nM) and rapamycin (30 nM) were used as positive (+) controls. * *p* > 0.05. Data on graph correspond to the mean ± standard error of the mean from at least three independent experiments.

Conventional May-Grumwald-Giemsa staining revealed interesting morphological characteristics, as numerous cellular rounded cytoplasm-containing vesicles surrounding the nucleus, resembling autophagosomes, appeared in MALME-3M cells treated with chromomycin A2 at 30 nM during 48 h (Figure 3a). In addition, a strong reduction of cell density was noticeable. Flow cytometry analysis showed a significant increase of internucleosomal fragmentation in cells exposed to chromomycin A2at 30 nM (Figure 3b). Analysis of relevant protein markers for autophagy and apoptosis by western blot supported the involvement, mostly, of the autophagic process following treatment of MALME-3M with chromomycin A2. The increased expression of beclin-1 was evident, when compared to control cells (Figure 3c). This protein has a central role in the regulation of autophagy in mammals, especially during autophagosome formation [39]. LC3 proteins also assist initial autophagosome formation through its conversion to a membrane-bound lipidated form by a pro-autophagic signal, which remains bound to the luminal membrane until fusion with lysosomes is completed [40]. Lipidation of LC3 family members, LC3-A and LC3-B, is an autophagosomal marker [41] and, in the present study, expression of both was clearly increased after treatment with chromomycin A2 at 10 and 30 nM (Figure 3c). Caspase 3 and PARP play crucial role in the execution-phase of apoptosis. The cleaved forms of Caspase 3 and PARP trigger their active state [42,43]. The treatment with chromomycin A2 had no obvious effect in the expression of the later proteins (Figure 3c). Although Caspase 3 and PARP were not altered by chromomycin A2, it is important to mention that generally autophagy is followed by apoptosis in cells exposed to anticancer agents [44]. In fact, many signal transduction pathways may regulate both processes, explaining the sequential nature of the cellular events [44]. As observed for cell viability, cell density and cell cycle progression, chromomycin A3 at 30 nM did not induce any evident alterations in treated cells.

### 2.4. Induction of Autophagy by Chromomycin A2

Autophagy is an intracellular catabolic pathway that involves the clearance or recycling of unessential or dysfunctional proteins and organelles. This process may promote cell survival by serving as an alternate energy source during starvation periods, thus maintaining homeostasis and cell viability [45].

**Figure 4:**
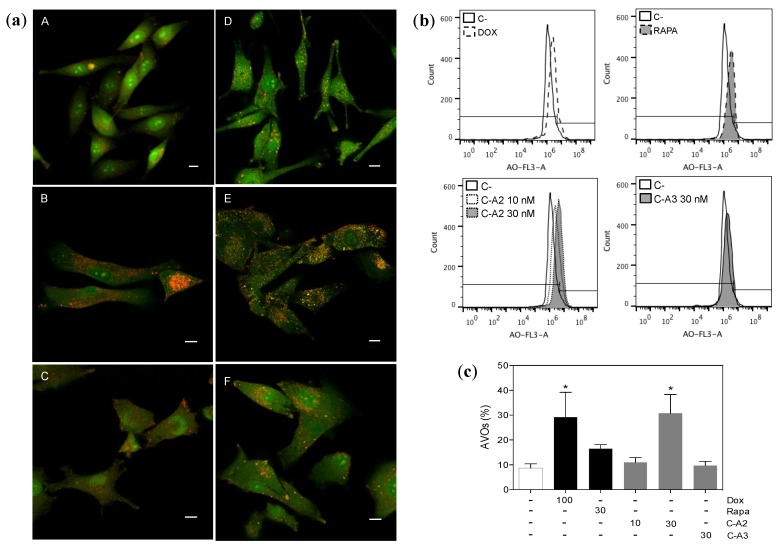
Chromomycin-A2 (**C-A2**) induces autophagy on MALME-3M cells. (**a**) Confocal images depicting the formation of acid vesicular organelles (AVOs) with double-staining with acridine orange (AO) on MALME-3M cells treated for 48h (A, DMSO; B, doxorubicin; C, rapamycin; D, chromomycin A2 10 nM; and E, chromomycin A2 30 nM; F, chromomycin A3, **C-A3**, 30 nM), horizontal bars = 10 μm; Representative histograms (**b**) and graph (**c**) of flow cytometry quantification of AVOs with AO staining. DMSO (0.4% v/v) was used as negative control (−) and doxorubicin (100 nM) and rapamycin (30 nM) were used as positive controls. * *p* > 0.05. Data on graph correspond to the mean ± standard error of the mean from at least three independent experiments.

The formation of autophagosomes is a key step for the cellular installation of the autophagic process. These are rounded vesicles with double-layer membranes that sequester a portion of cytoplasm (with protein and organelles to recycle) to be later delivered to lysosomes, for digestion by acid hydrolases [45]. Thus, for recognition of this acidic compartment, acridine orange was used due to its propriety of moving across biological membranes when uncharged. Its protonated form, on the other hand, accumulates in the acidic vacuolar organelles (AVOs) and fluoresces in an orange tone [46]. Staining of MALME-3M cells treated with chromomycin A2 at 30 nM revealed the appearance of numerous AVOs, comparable to doxorubicin, shown by concentration of the dye in the vesicles (Figure 4). Rapamycin, recognized as a classical autophagy inductor, did not induce a significant accumulation of AVOs. It is important to mention that autophagy is an orchestrated cascade of events with a time course that can be variable dependent on the stimulus.

Although chromomycin A2 has not been studied to the extent of chromomycin A3, no evidences depicting the induction of autophagy by any of the aureolic acids have been reported. However, it is not unlikely that starvation conditions, generated by the deprivation of nutrients as a consequence of reduced transcription activity, may be connected to the observations emphasized herein. The association between these two processes should be further addressed in order to establish causality of the relationship; however, the induction of autophagy might influence the way by which the biological activities of aureolic acids are engaged in a clinical panorama from now on.

## 3. Experimental Section

### 3.1. General Experimental Procedures

NMR spectra were obtained in a a Bruker Avance III spectrometer (500 and 125 MHz for ^1^H and ^13^C NMR, respectively) equipped with a 1.7 mm TCI MicroCryoProbe™ (Bruker Biospin, Fällanden, Switzerland). Chemical shifts were reported in ppm using residual CDCl_3_ (δ 7.27) as internal reference. LC-MS analysis was performed on an Agilent 1100 single Quadrupole (Agilent Tehcnologies, Santa Clara, CA, USA), while the dereplication was carried using in house and commercial databases (Chapman & Hall Dictionary of Natural Products). HRESIMS spectra were acquired using a Bruker maXis QTOF (Bruker Daltonik GmbH, Bremen, Germany) mass spectrometer coupled to an Agilent 1200 LC (Agilent Technologies, Waldbronn, Germany) UV spectra were obtained with an Agilent 1200 DAD. Preparative and semipreparative HPLC was performed using a Gilson GX-281 system (Gilson Inc., Middleton, WI, USA).

### 3.2. Isolation and Identification of Bacterial Strain

Bacterial strain (previously identified as BRA-090) was recovered from a marine sediment sample collected at Paracuru Beach [03°23'S; 39°54'W], located on the west coast of Ceará State, on the northeastern region of Brazil. Freshly collected sediment was left to air dry under a flow hood during 48h. Using a sterile sponge plug, dry sediment was serially stamped onto Petri dishes containing trace mineral agar (0.1% glucose, 0.1% yeast extract, K_2_HPO_4_, Na_2_HPO_4_, KNO_3_, NaCl, MgSO_4_·7H_2_O, CaCl_2_·2H_2_O and 18% agar in 75% seawater, and added a solution containing FeSO_4_·7H_2_O, ZnSO_4_·7H_2_O, MnSO_4_·4H_2_O, CuSO_4_·5H_2_O, CoSO_4_·7H_2_O, H_3_BO_3_ and (NH_4_)_6_Mo_7_O_24_·4H_2_O), supplemented with 0.1 mg/mL cycloheximide (Sigma Aldrich, Saint Louis, MI, USA), and incubated at 28 °C. After nearly 4 weeks, an individualized colony of the strain was transferred to a new dish layered with A1 agar (10% soluble starch, 4% yeast extract, 2% peptone and 18% agar in 75% filtered sea water) for purification.

For taxonomic identification of the strain, a molecular based approach was carried out. Microbial genomic DNA was extracted from a pure culture of the bacteria using a method adapted from that of [47]. An aliquot from fresh bacterial culture grown during 4 days was centrifuged (215 g; 3 min) to remove excess culture medium, then added to 1 mL of lysis buffer [2% (m/v) CTAB, 1.4 M NaCl, 20 mM EDTA, 100 mM Tris-HCl (pH 8.0)], freshly added with 5 µg proteinase K (v/v; Invitrogen) and 0.5% 2-mercaptoethanol. Sample was frozen at −80 °C for 3–5 min and soon after thawed in a dry bath for 3 min at 65 °C; this procedure was repeated two times. Next, an equal volume of the solution was added to phenol:chloroform:isoamyl alcohol (25:24:1; v/v), homogenized and centrifuged (7 min; 9520 g; 4 °C). The aqueous phase was recovered and mixed with 400 µL of chloroform, then centrifuged (5 min; 9520 g; 4 °C). Genomic DNA was precipitated by addition of 0.75× volume of ammonium acetate and 1 volume of ice-cold isopropyl alcohol, followed by centrifugation (30 min, 9520× *g*, 4 °C). The supernatant was discarded; the pellet was washed with ice-cold ethanol (70%), centrifuged (10 min; 13,709× *g*; 4 °C) and dissolved in 35–50 µL of TE buffer (10 mM Tris-HCL e 1 mM de EDTA) overnight. Genomic DNA was quantified using a microvolume spectrophotometer (Thermo Scientific; NanoDrop 2000c, Wilmington, NC, USA).

Amplification of the 16S rRNA gene was done in a total volume of 25 µL containing PCR mix (AmpliTaq Gold^®^ 360 Master Mix, Foster City, CA, USA), 0.1 nM of each primer, 27F (5′-AGAGTTTGATCCTGGCTCAG-3′) and 1492R (5′-TACGGCTACCTTGTTACGACTT-3′), and 100–150 ng of DNA. PCR was initiated with a denaturation step at 94 °C for 12:30 min followed by 35 cycles of the following protocol: denaturation at 94 °C for 1 min, extension at 72 °C for 1 min and annealing at 63 °C for 1 min. For the last cycle, 7 min of extension at 72 °C was performed. PCR products were visualized by electrophorese on 1% agarose gels stained with SYBR safe and Blue Juice. PCR products were purified by mixing 5 µL of PCR reaction product with 2 µL of ExoSAP-IT (Affymetrix Inc., Cleveland, OH, USA), then incubating samples at 37 °C for 15 min, followed by a second incubation at 80 °C for 15 min. DNA sequencing was performed on an Applied Biosystems 3500 Genetic Analyzer using the ABI PRISM BigDyeTM Terminator Cycle Sequencing kit (Applied Biosystems, Foster City, CA, State, USA) and the same primers used for amplification, following the protocols supplied by the manufacturer.

The obtained forward and reverse sequences were aligned and the contig generated with assistance of the software Geneious 7 (Biomatters Ltd., Auckland, New Zealand) and compared to sequences within the NCBI database (http://www.ncbi.nlm.nih.gov/) using the BLAST.

### 3.3. Up-Scale Bacterial Growth and Isolation of Chromomycin A2

*Streptomyces* sp. was grown in A1 broth (10% soluble starch, 4% yeast extract and 2% peptone) supplemented with 0.1% calcium carbonate, and solutions of Fe_2_(SO_4_)_3_ and KBr, in 75% filtered sea water. The culture broth (14 L) was fermented during 7 days under 150 rpm agitation at 28 °C, and extracted with EtOAc (1:1). The solvent was removed under reduced pressure to yield 570 mg of crude extract. The dried extract was subjected to reversed phase flash chromatography (RediSep C_18_, gradient H_2_O:MeOH from 20% to 100% MeOH in 12.5 min + 100% MeOH for 15 min, flow rate 10 mL/min, UV detection at 210 and 279 nm) to obtain 15 fractions (F1–F15) of approximately 18 mL each. Subfraction F8 was analyzed by LC-HRESIMS employing a Zorbax SB-C_8_ column (2.1 × 30 mm) and a gradient elution profile of 10% B (90% CH_3_CN, 10% H_2_O, 1.3 mM TFA, 1.3 mM ammonium formiate)/90% A (10% CH_3_CN, 90% H_2_O, 1.3 mM TFA, 1.3 mM ammonium formiate) to 100% B in 6 min, kept at 100% B for 2 min and returned to 10% B for 2 min at flow of 300 µL/min. Peaks with *m/z* 1211.5476, 1183.5164 and 1197.5310 were identified as chromomycin A2 (**1**), chromomycin A3 (**2**) and demethylchromomycin A2 (**3**), respectively. Subfraction F8 was subjected to preparative HPLC (Zorbax C_8_, 2.1 × 250 mm, gradient H_2_O:CH_3_CN from 30% to 70% CH_3_CN in 35 min, flow rate 20 mL/min, UV detection at 210 and 280 nm) to yield 80 fractions (F1–F80). Fraction F33 was further purified using semipreparative HPLC (Zorbax C_18_, 9.4 × 250 mm, gradient H_2_O/CH_3_CN from 50% to 56% CH_3_CN in 35 min, flow rate 3.6 mL/min, UV detection at 210 and 280 nm) to yield the bioactive pure compound **1** (1.0 mg).

Chromomycin A2 (**1**): yellow amorphous solid; UV (MeOH) λ_max_ 279 nm; ^1^H NMR spectrum (CDCl_3_): δ 1.22 (d, *J* = 7.0 Hz), 1.23 (d, *J* = 7.0 Hz), 1.25 (d, *J* = 6.5 Hz), 1.30 (t, *J* = 5.7 Hz), 1.36 (s), 1.37 (d, *J* = 4.4 Hz), 1.39 (t, *J* = 6.7 Hz), 1.72 (m), 1.76 (m), 2.06 (m), 2.18 (s, CH_3_), 2.19 (s, CH_3_), 2.30 (dd, *J* = 13.3 and 5.4 Hz), 2.49 (dd, *J* = 12.0 and 5.4 Hz), 2.62 (m), 2.65 (m), 2.70 (m), 3.13 (m), 3.23 (d, *J* = 2.6 Hz), 3.32 (m), 3.39 (m), 3.52 (s, OCH_3_), 3.61 (s, OCH_3_), 3.82 (q, *J* = 6.5 Hz), 3.89 (q, *J* = 6.5 Hz), 4.01 (m), 4.23 (s), 4.37 (q, *J* = 6.5 Hz), 4.61 (d, *J* = 9.4 Hz), 4.72 (d, *J* = 2.7 Hz), 5.03 (sl), 5.10 (d, *J* = 9.9 Hz), 5.13 (s), 5.18 (sl), 5.23 (d, *J* = 9.7 Hz), 6.65 (s), 6.76 (s), 9.82 (s), 15.7 (s); HRESIMS *m*/*z* 1211.5476 [M + H]^+^ (calcd. for C_59_H_87_O_26_, 1211.5480). 

### 3.4. Cytotoxicity Assay

Cytotoxic activity of **1** was evaluated against six different human tumor cell lines, HCT-116 (colon adenocarcinoma), HL-60 (leukemia), MALME-3M (metastatic melanoma), OVCAR-8 (ovarian carcinoma), PC-3M (metastatic prostate carcinoma) and SF-295 (glioblastoma), and a human non-tumor cell line MRC-5 (fetal lung fibroblast). MALME-3M cells were maintained in IMDM medium supplemented with 20% fetal bovine serum (v/v), 100 U/mL penicillin and 100 µg/mL streptomycin, and the other cell lines were maintained in RPMI 1640 medium supplemented with 10% fetal bovine serum (v/v), 2 mM glutamine, 100 U/mL penicillin and 100 µg/mL streptomycin. All cells were kept at 37 °C under a 5% CO_2_ atmosphere. Cell cultures were regularly split to keep them in a logarithmic growth phase. IC_50_ values were determined using the MTT assay [48]. Cells were plated into 96-well plates (3 × 10^5^ cells/mL for suspended leukemia cells and 5 × 10^4^ cells/mL for adherent solid tumor cells). Adherent cells were plated 24 h prior to addition of C-A2 and incubated for 24, 48 or 72 h. Negative controls received DMSO, whereas positive controls received doxorubicin. Three hours before the end of the incubation periods, 150 µL of a stock solution (0.5 mg/mL) of MTT (3-(4,5-dimethyl-2-thiazolyl)-2,5-diphenyl-2H-tetrazolium bromide) was added to each well, and, 3 h later, removed. Absorbance was measured using a multiplate reader (Fisher Scientific, model Multiskan FC, Hampton, VI, USA). The effect was quantified as the percentage of the control absorbance at 595 nm and IC_50_ values, along with 95% confidence intervals, were calculated by non-linear regression using GraphPad Prism 4.0 (Intuitive Software for Science, La Jolla, CA, USA).

### 3.5. Cell Viability Analyses

MALME-3M cells were seeded in a 24-well plate (5 × 10^4^ cells/mL) and treated with **1** at 10 or 30 nM for 48 h along with 0.4% DMSO (v/v) or 100 nM doxorubicin, as negative and positive controls, respectively. Cells were harvested with trypsin-EDTA 0.05% (Gibco), centrifuged and resuspended in a 5 µg/mL propidium iodide (PI) solution (Sigma Aldrich). After 5 min incubation in the dark, five thousand events were acquired using the Accuri C6 flow cytometer (BD Biosciences, Franklin Lakes, NJ, USA), on a gated region to exclude debris and doublets from the analysis (supplementary Figure S5). The experiments were repeated at least three times. The differences between negative control and experimental groups were determined by analysis of variance (ANOVA) followed by Dunnett’s test on GraphPad Software 4.0 (Intuitive Software for Science, La Jolla, CA, USA). The minimal significance level was set at *p* < 0.05.

### 3.6. Cell Cycle and DNA Fragmentation Analyses

MALME-3M cells seeded in a 24-well plate (5 × 10^4^ cells/mL), were treated with **1** at 10 or 30 nM, or controls. After 48 h exposure period, cells were collected from the plate with trypsin-EDTA 0.05% (Gibco, Saint Louis, MI, USA), centrifuged and resuspended in a solution containing 5 µg/mL PI, 0.1% citrate and 0.1% Triton X-100. After 30 min incubation in the dark, five thousand events were acquired on the Accuri C6 flow cytometer (BD Biosciences, Franklin Lakes, NJ, USA) set with gated regions to exclude debris and doublets from the analysis (Supplementary Figure S6). Cell cycle histograms were acquired with the FL3 channel set in linear scale. Cell cycle profile was then analyzed with the ModFit LT 3.3 software (Verity Software House, Topsham, ME, USA) to determine the respective percentages of cells in G1/G0, S and G2/M phases. Moreover, sub-G1/G0 cells, which relates to those undergoing DNA fragmentation and loss, were also accounted in the cell cycle profile analysis. The experiments were repeated at least three times. Differences between negative control and experimental groups were determined by analysis of variance (ANOVA) followed by Dunnett’s test using GraphPad Software 4.0 (Intuitive Software for Science, La Jolla, CA, USA). The minimal significance level was set at *p* < 0.05.

### 3.7. Morphological Analysis

MALME-3M cells were seeded (5 × 10^4^ cells/mL) in 24-well plates pre-planted with sterile coverslips and treated with **1** at 10 or 30 nM **1**, or controls. After 48 h exposure, cells were fixed and stained using a kit for fast staining of hematologic cells (Quick Panoptic Kit, Laboclin, Paraná). Untreated or treated cells were examined for morphological changes under light microscopy (Olympus, model BX-41, Miami, FL, USA).

### 3.8. Detection and Quantification of Acidic Vesicular Organelles (AVOs)

Detection of AVOs was conducted by treating MALME-3M cells (3 × 10^4^ cells/mL) in a 35 mm glass-bottom culture dish (MatTek Corporation, Ashland, OR, USA) with 10 and 30 nM **1**, or controls, for 48 h. Next, acridine orange (AO) was added at a final concentration of 1 µg/mL for a period of 15 min in the dark at 37 °C [46] and images were obtained on an inverted confocal microscope with a Plan-APOCHROMAT^®^ 63×/1.40 objective (LSM 710, Zeiss, Oberkochen, Germany).

To quantify the development of AVOs, cells were seeded in a 24-well plate (5 × 10^4^ cells/mL), and treated as described above. After 48 h treatment, cells were harvested with trypsin-EDTA 0.05% (Gibco, Saint Louis, MI, USA), centrifuged and resuspended in a 1 µg/mL AO solution. After 15 min incubation in the dark at 37 °C, cells were washed with Dulbeccos’s phosphate buffered saline (PBS). Then five thousands events were acquired in a gated region set in a FSC *vs*. SSC dot plot graph to exclude debris using Accuri C6 flow cytometer and Accuri C6 software (BD Biosciences, Franklin Lakes, NJ, USA). AVOs were detected in a gate set in high fluorescence level of FL3 channel. The experiments were repeated at least three times. The differences between negative control AVOs and experimental groups were determined by analysis of variance (ANOVA) followed by Dunnett’s test using GraphPad Software 4.0 (Intuitive Software for Science, La Jolla, CA, USA). The minimal significance level was set at *p* < 0.05.

### 3.9. Western Blot

MALME-3M cell were seeded in a 6-well plate (5 × 10^4^ cells/mL) and treated with **1** at 10 or 30 nM, or controls, for 48 h. Suspended cells were collected, centrifuged, washed with PBS, resuspended with 150 µL of radioimmunoprecipitation assay (RIPA) buffer (Sigma Aldrich，Saint Louis, MI, USA) and returned to the plate for collection of adhered cells, which were previously washed with PBS. After 20 min in an ice-cold recipient, cells were transferred to a 1.5 mL tube, lysed by sonication for 10s and centrifuged at 13,700× *g*. The supernatant was collected and the protein concentration therein was determined by a modified Lowry analysis (BioRad Laboratories, Hercules, CA, USA) in comparison with bovine serum albumin (BSA) standards and analyzed on GraphPad Prism 4.0. Samples containing 10 µg of total protein extract from each treatment were diluted with 2× Laemmlli buffer (Bio-Rad, Hercules, CA, USA) and loaded onto a 5%–12.5% gradient SDS-PAGE gel placed in Tris-Glycine-SDS buffer (25 mM Tris, 192 mM glycine, 0.1% SDS (w/v), pH 8.3). After running the electrophoresis for nearly 2 h 40 min at 120 V and 40 mA, proteins were then transferred from the gel onto a Hybond-P PVDF membrane (GE Healthcare, Little Chalfont, UK) in Tris-glycine-MeOH transfer buffer (25 mM Tris, 192 mM glycine, 20% MeOH (v/v); BioRad Laboratories, Hercules, CA, USA) at 300 mA and 100 V for 1 h 20 min. The membrane was blocked for 30 min with 5% non-fat dried milk (w/v) in tris-buffered saline (TBS) and then washed 3 times in TBS-T (137 mM NaCl, 20 mM Tris, 0.1% Tween 20 (w/v), pH 7.6). Next, the membrane was incubated overnight, at 4 °C, with the ascribed primary antibody diluted at 1:1000 in BSA 5% in TBS (137 mM NaCl, 20 mM Tris-HCl, pH 7.6). The membrane was then washed 3 times with TBS-T, incubated for 1 h in room temperature with the appropriate alkaline phosphatase linked secondary antibody diluted to 1:2000 in 5% non-fat dried milk (w/v) in TBS. The membrane was washed with TBS-T and developed using a 1-step NBT/BCIP solution (Sigma-Aldrich, Saint Louis, MI, USA). The following primary antibodies were used, cyclin A2, cyclin D1, cyclin D3, LC3-A, LC3-B, beclin-1, caspase-3, caspase-3 cleaved, PARP, cleaved PARP and β-actin, as a loading control. Anti-rabbit IgG and anti-mouse IgG were used as secondary antibodies. All antibodies were obtained from Cell Signaling Technology, Inc. (Beverly, MA, USA).

## 4. Conclusions

The present work described the autophagy-inducing effects of chromomycin A2 in a metastatic melanoma cell line. Biochemical evidences collected experimentally to support this statement include increased expression of beclin-1 and lipidation of the autophagosomal markers LC3-A and LC3-B, while morphological evidences are depicted by the appearance of acidic vacuoles organelles, as well as numerous cellular rounded cytoplasm-containing vesicles surrounding the nucleus, resembling autophagosomes. Although chromomycins are well known for their anticancer activity, this is the first report of autophagy induction by this class of compounds. 

## Supplementary Files

Supplementary File 1
