# Supplementary material for: Chromomycin A2 Induces Autophagy in Melanoma Cells
Source: Mar Drugs. 2014 Dec 4;12(12):5839–55. doi: 10.3390/md12125839 (PMC4278204; doi:10.3390/md12125839)
Supplement: Supplementary File 1 [file marinedrugs-12-05839-s001.pdf]

Supplementary Information

Table of Contents

Figure S1 ^{1}H NMR spectrum for chromomycin A2 (**1**) in $CDCl_3$.

Figure S2 ^{1}H NMR spectrum expansion for chromomycin A2 (**1**) in $CDCl_3$.

Figure S3 HR-ESI-MS spectrum of chromomycin A3 (**2**).

Figure S4 UV spectrum of chromomycin A3 (**2**).

Figure S5 Cell viability analysis of MALME-3M after 48h treatment. The gating strategy for flow cytometry analysis is presented as follows. All cells were identified using SSC-A *vs.* FCS-A and doublets excluded using FSC-A *vs.* FSC-H. Non-viable cells show high PI fluorescence in FL3 channel (red fluorescence). Cell density was measured using an additional gate to fit normal cell morphology.

Figure S6 Cell cycle analysis of MALME-3M treated with 10 and 30 nM **1** for 48h. (**a**) Gating strategy for flow cytometry analysis. Cells were identified using SSC-A *vs.* FCS-A and gated for singlets using PI-H *vs.* PI-A; (**b**) Representative histograms of each treatment analyzed and modeled by Modfit LT 4.0 program. DMSO (0.4% v/v) and doxorubicin (100 nM) were used, respectively, as negative (−) and positive (+) controls.

Table S1 Cytotoxicity of doxorubicin on select cell lines. Activity of doxorubicin was evaluated using the MTT assay after 72 h incubation. The IC_{50} [µM] values and 95% CI were obtained via nonlinear regression.

Figure S1. ^{1}H NMR spectrum for chromomycin A2 (**1**) in $CDCl_3$.

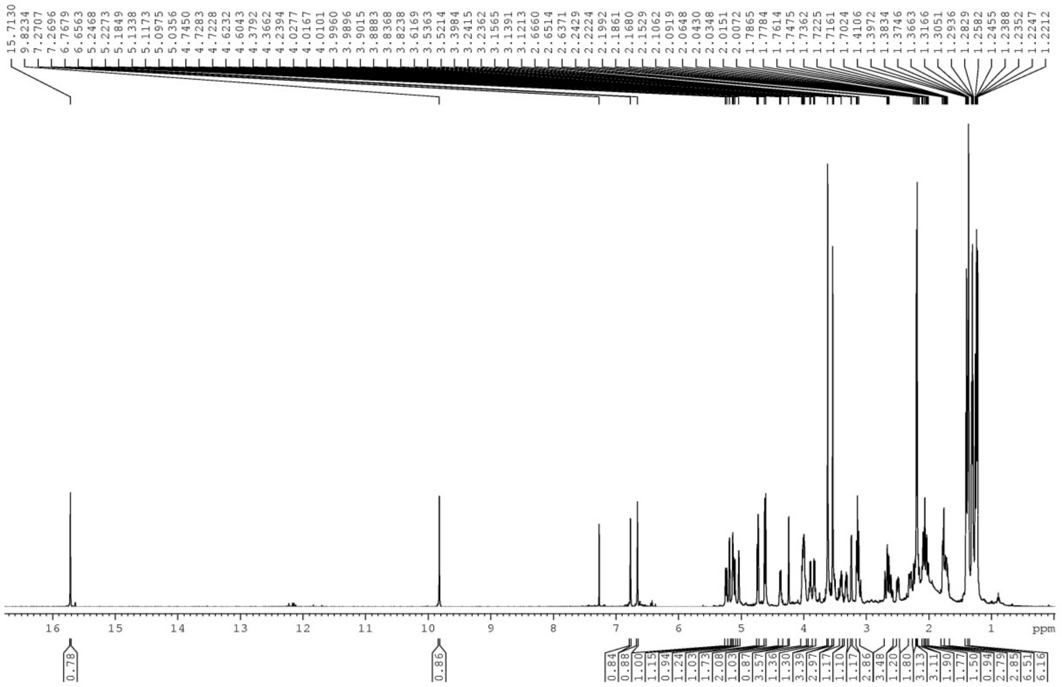

Figure S2. ^{1}H NMR spectrum expansion for chromomycin A2 (**1**) in $CDCl_3$.

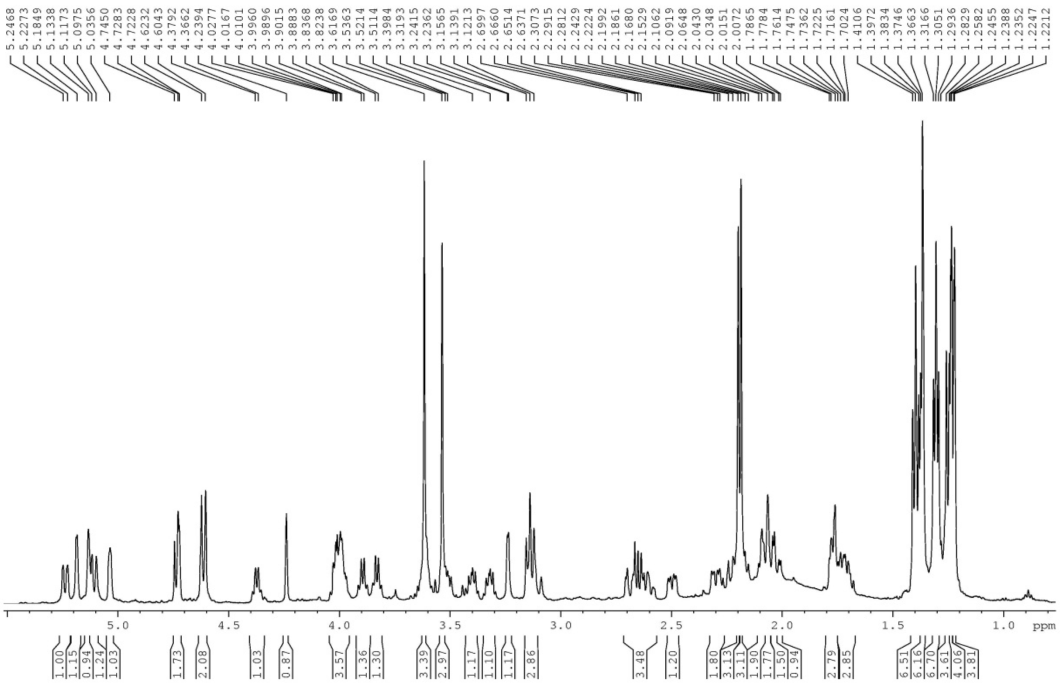

S1. Isolation of Chromomycin A3

Attempting to isolate compound **2** in order to compare the results with the ones obtained with compound **1**, the bacterial strain was regrown again under the same conditions. The culture broth (21.9 L), after fermentation, was extracted with EtOAc and subsequently the solvent was removed under reduced pressure to yield 708.6 mg of crude extract. This extract was subjected to a silica gel chromatography column using the solvents hexane/CH_2Cl_2 1:1, CH_2Cl_2, CH_2Cl_2/EtOAc 1:1, EtOAc, EtOAc/MeOH 8:2, EtOAc/MeOH 1:1 and MeOH to yield fractions A – G. Fractions D and E were combined, since both showed similar cytotoxicity and chemical profile by TLC analysis. Fraction D/E (248.6 mg) was chromatographed on SPE C-18 cartridge (Strata C18-E, 20 g/60 mL, 55 µm, 70 Å), eluting with H_2O/MeOH 8:2, 6:4, 4:6, 2:8, MeOH and EtOAc/MeOH 1:1. After cytotoxic assays and TLC analysis, the three last fractions were combined and rechromatograped on silica gel employing the solvents CH_2Cl_2/MeOH 9:1, 8:2, 7:3 1:1 and MeOH. Fraction CH_2Cl_2/MeOH 7:3 (6.4 mg) was subjected to semipreparative HPLC (Phenomenex C_{18} 4.6 × 250 mm, gradient H_2O/CH_3OH from 60% to 100% CH_3OH in 20 min, flow rate 4.7 mL/min, UV detection 210 – 400 nm) to yield compound **2** (t_R 14.65, 0.4 mg). Compound **2** was identified by HRESIMS, UV data and AntiMarin data base as chromomycin A3 (**2**) λmax 413.87, 277.53 and 228.76 nm; HRESIMS *m/z* 1205.4865 $[M + Na]^+$).

Figure S3. HR-ESI-MS spectrum of chromomycin A3 (**2**).

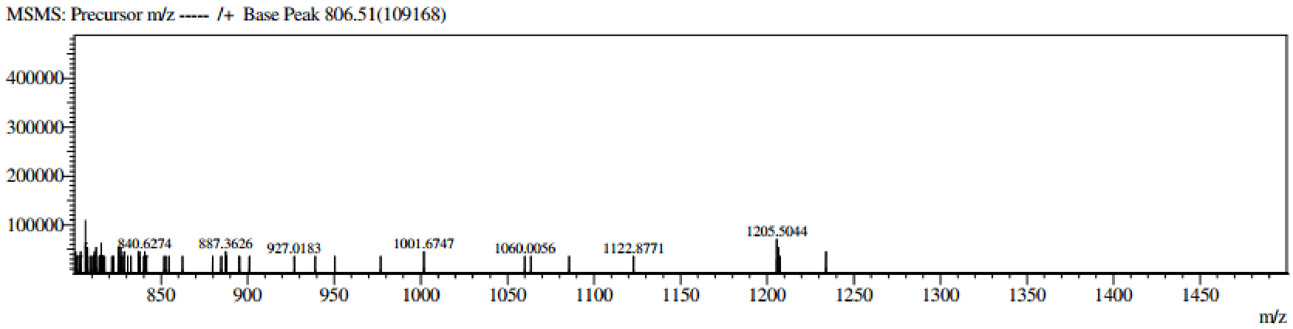

Figure S4. UV spectrum of chromomycin A3 (**2**).

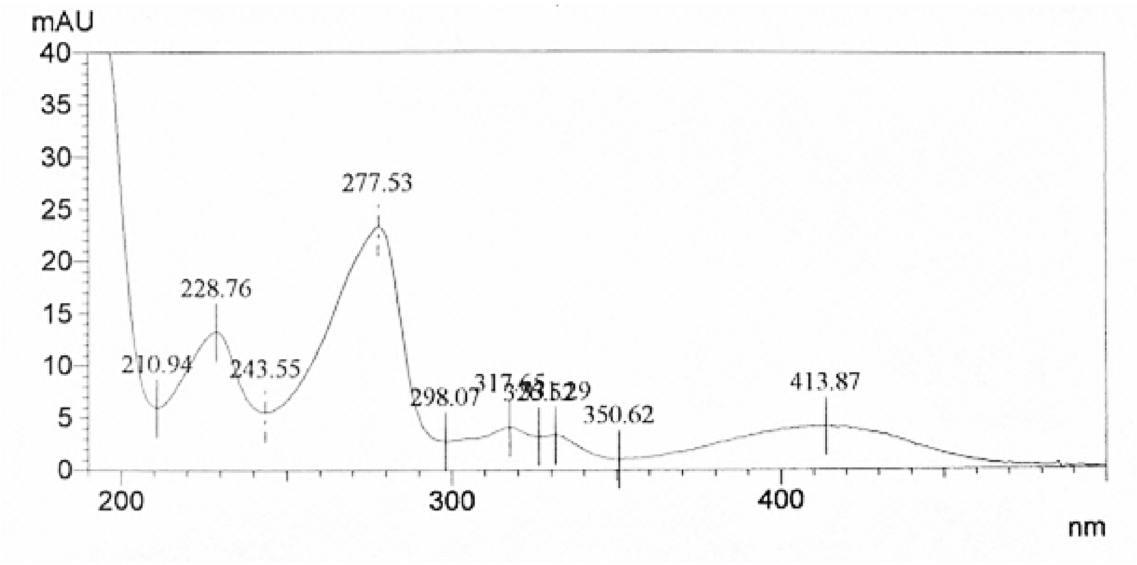

Figure S5. Cell viability analysis of MALME-3M after 48h treatment. The gating strategy for flow cytometry analysis is presented as follows. All cells were identified using SSC-A *vs.* FCS-A and doublets excluded using FSC-A *vs.* FSC-H. Non-viable cells show high PI fluorescence in FL3 channel (red fluorescence). Cell density was measured using an additional gate to fit normal cell morphology.

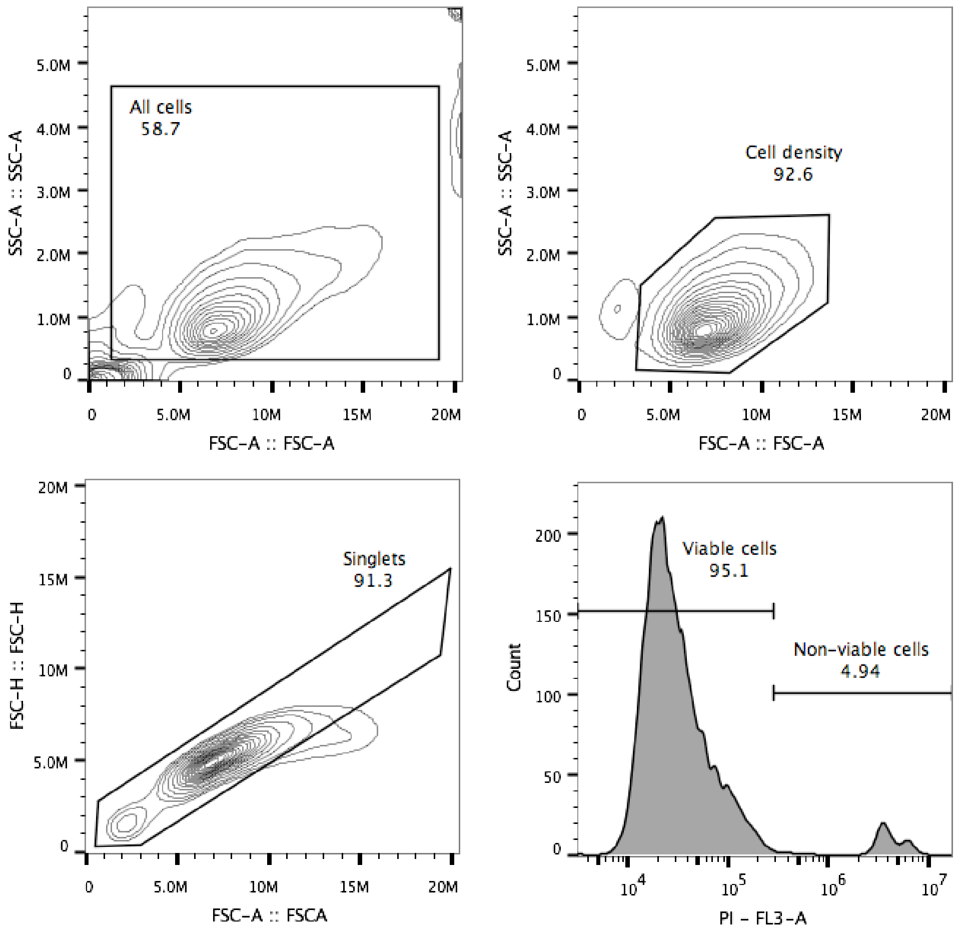

Figure S6. Cell cycle analysis of MALME-3M treated with 10 and 30 nM **1** for 48h. (**a**) Gating strategy for flow cytometry analysis. Cells were identified using SSC-A *vs.* FCS-A and gated for singlets using PI-H *vs.* PI-A; (**b**) Representative histograms of each treatment analyzed and modeled by Modfit LT 4.0 program. DMSO (0.4% v/v) and doxorubicin (100 nM) and rapamycin (30 nM) were used, respectively, as negative (−) and positive (+) controls.

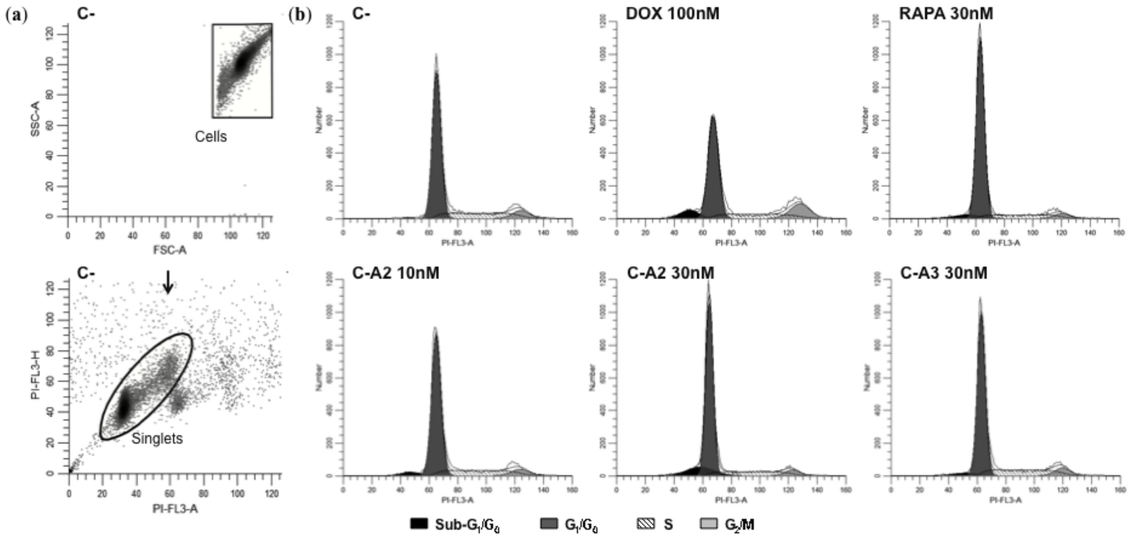

Table S1. Cytotoxicity of doxorubicin on selected cell lines. Activity of doxorubicin was evaluated using the MTT assay after 72 h incubation. IC_{50} [nM] values and 95% CI were obtained via nonlinear regression.

**Cell Line**	**IC_{50} [nM] (CI 95%)**
	**72 h**
HCT-116	42 (30–59)
HL-60	6 (4–8)
MALME-3M	244(179–334)
OVCAR-8	36(25–50)
PC-3M	0.9922 (0.5204–1.892)
SF-295	26 (18–36)
MRC-5	3828 (2105–6960)

© 2014 by the authors; licensee MDPI, Basel, Switzerland. This article is an open access article distributed under the terms and conditions of the Creative Commons Attribution license (http://creativecommons.org/licenses/by/4.0/).
